# Psychotropic prescribing trends in a Peruvian General Hospital during and after the COVID-19 pandemic: an interrupted time-series analysis

**DOI:** 10.3389/fpubh.2026.1892034

**Published:** 2026-10-09

**Authors:** José Salvador-Carrillo, Ana Carolina Figueiredo-Modesto, Luciane Cruz-Lopes, Claudio Flores-Flores, Joseph Pinto, Jeff Huarcaya-Victoria

**Affiliations:** 1 Postgraduate Program in Pharmaceutical Sciences–University of Sorocaba, São Paulo, Brazil; 2 Department of Human Medicine, Universidad Privada San Juan Bautista, Lima, Peru; 3 Unidad de Investigación Básica y Traslacional, Auna Ideas, Lima, Peru; 4 Psychiatry Research Unit, Department of Psychiatry, Hospital Nacional Guillermo Almenara Irigoyen, Lima, Peru

**Keywords:** antidepressants, COVID-19, drug utilization, interrupted time-series analysis, Peru, psychotropic drugs

## Abstract

**Purpose:**

To evaluate trends in psychotropic prescription rates (PRs) in a Peruvian general hospital during and after the COVID-19 pandemic.

**Materials and methods:**

We conducted an interrupted time-series (ITS) study using routinely collected clinical data from hospitalized patients with psychiatric diagnoses between September 2020 and December 2024. Psychotropic PRs, defined as the number of weekly psychotropic prescriptions per 1,000 psychiatric consultations, were calculated overall and by subclass: antidepressants (N06A), antipsychotics (N05A), anxiolytics (N05B), and mood stabilizers (N05AN01/N03A). For ITS analysis, the study period was divided into six predefined phases (two waves, three relaxation phases, and one post-pandemic phase) based on COVID-19 mortality and transmission trends in Peru. Segmented regression models were applied to estimate phase-specific level and slope changes.

**Results:**

A total of 6,151 patients were included. The overall psychotropic PR was higher during the post-pandemic period compared with the pandemic period (1,308 vs. 1,254; *p* = 0.023). ITS analyses showed a significant downward trend in overall psychotropic prescribing during phase 1 (post-first wave stabilization, β = −25.53; 95% CI = −43.82 to −7.01; *p* = 0.007), followed by a structural break in phase 2 (second pandemic wave) with an abrupt level increase (β = 295.53; 95% CI = 60.48 to 530.57; *p* = 0.014) and slope reversal (β = 23.79; 95% CI = 3.33 to 44.26; *p* = 0.016), after which prescribing stabilized. A similar temporal pattern was observed for antidepressant PR. Anxiolytic PR displayed a transient upward slope change in phase 2 (β = 14.15; 95% CI = 1.80 to 26.50; *p* = 0.01), whereas antipsychotic and mood stabilizer prescribing remained stable across phases.

**Conclusions:**

Overall psychotropic prescribing showed phase-specific disruption followed by structural rebound and subsequent stabilization across the COVID-19 and post-pandemic periods. These findings provide real-world evidence and underscore the need to monitor psychiatric prescribing and mental health service adaptation in middle-income settings.

## Introduction

The COVID-19 pandemic led to substantial disruption to the population's mental health and exerted profound impacts on health care systems worldwide. The early years of the pandemic were associated with a 27.6% increase in cases of major depressive disorder and a 20.5% increase in anxiety disorders (1). Concurrently, the widespread implementation of public health measures, such as lockdowns, social distancing, and border closures, constrained access to mental health services and psychotropic medications (2–4). Moreover, this impact was more pronounced in low-income countries (LICs), where pre-existing socioeconomic inequalities and vulnerabilities in mental health care systems intensified the effects of the pandemic (5).

Peru was among the countries most severely affected by the COVID-19 pandemic, with one of the highest mortality rates worldwide (approximately 6,650 deaths per million population) (6). Increases in depressive, anxiety, and distress symptoms were consistently reported during this period (7–9), coinciding with substantial disruptions to mental health service delivery, including reduced patient visits and worsened inequalities in access to care among vulnerable populations (10, 11). However, these studies were conducted using symptom-based surveys (7–9, 12), and may not accurately represent how the healthcare system responds in clinical practice.

Evidence suggests a heterogeneous pattern of psychotropic medication use across the COVID-19 pandemic, with changes in prescribing behavior by drug class and health system context (13). Some studies showed a significant increase in prescription of antidepressants and mood stabilizers during the early phases of the pandemic (14, 15), often attributed to psychological distress and expanded use of remote consultations. In contrast, abrupt declines in anxiolytic and antipsychotic prescribing were observed in some regions of Europe (16, 17) and Australia (18), likely reflecting disruptions in health care access and reduced availability of in-person services. Overall, these variations reflect the complex interplay of factors, highlighting the importance of monitoring these patterns, particularly in more vulnerable health care settings (19).

Despite extensive research on the mental health impact of the COVID-19 pandemic, several limitations and regional knowledge gaps persist. Most available studies come from high-income countries (HICs) (13), leading to a paucity in comprehensive data from middle-income countries (MICs) and LICs and how the pandemic affected more vulnerable health care systems. Moreover, several works rely on aggregated prescription or sales data, providing limited insights into clinical populations or a specific level of care (20, 21). Evidence from Latin America is scarce, and hospital-based psychiatric settings in countries such as Peru are absent from the literature. To address these gaps, this study aimed to evaluate temporal trends in psychotropic prescribing across predefined COVID-19 pandemic phases using an interrupted time-series (ITS) analysis of routinely collected data from a Peruvian general hospital. Our findings provide real-world evidence from an underrepresented Latin American setting.

## Materials and methods

### Study design

We conducted a retrospective, single-center, and ITS study. Findings were reported following the recommendations of the RECORD-PE (22).

### Setting and data source

This study utilized a database from the Hospital Nacional Guillermo Almenara Irigoyen (HNGAI), located in Lima, a tertiary-level general hospital belonging to the Peruvian Social Security system (EsSalud). The hospital has an approximate capacity of 960 beds and played a central role in the response to the COVID-19 pandemic in Peru.

The database was generated from routine psychiatric evaluations by the Consultation-Liaison Psychiatry Unit (CLPU). This unit provided specialized mental health care to hospitalized patients referred from different medical and surgical services. The database includes sociodemographic characteristics, psychiatric diagnosis before hospital admission, and those established during hospitalization (coded using ICD-10), and psychotropic medications prescribed during their stay.

The dataset was extracted and made available for research in January 2025 and included routine psychiatric assessments performed between September 2020 and December 2024.

### Study population

The inclusion criteria were hospitalized patients who received at least one psychiatric consultation by the CLPU during the study period, aged 18 years or older, and had a documented psychiatric diagnosis based on ICD-10. Diagnoses could have been documented before or established during hospitalization. Patients with unverified psychiatric diagnoses were excluded.

### The COVID-19 pandemic phases in Peru

We divided the COVID-19 pandemic in Peru *a priori* into six different phases: two waves of the COVID-19 pandemic, three relaxation phases, and a post-pandemic phase. Each of these phases was defined based on the mortality and transmission trends in Peru, as reported by the Johns Hopkins University Coronavirus Resource Center (23) (Table 1 and Figure 1).

**Table 1:** Phases of the COVID-19 Pandemic in Peru during the period 2020 and 2024.

Phase	Phase name	Start week	End week	Short description
1	Post–first wave stabilization	W36 (2020)	W53 (2020)	Period following Peru's first COVID-19 wave, characterized by declining/stabilizing cases and deaths, partial relaxation of restrictions, and sustained healthcare pressure; used as an internal baseline within the study window.
2	Second wave resurgence	W01 (2021)	W33 (2021)	Period covering the resurgence and full development of Peru's second wave, including sustained increases in cases and deaths, healthcare system saturation, oxygen supply constraints, and high mortality burden.
3	Post–second wave stabilization and vaccination scale-up	W34 (2021)	W52 (2021)	Period following the decline of the second wave, marked by progressive epidemiological stabilization, expansion of vaccination coverage, gradual recovery of services, and lower mortality relative to earlier phases.
4	Omicron wave	W01 (2022)	W14 (2022)	Period dominated by the Omicron surge, with rapid increases in cases, high transmissibility, and comparatively lower severity/mortality per case, potentially affecting healthcare delivery and prescribing.
5	Post-Omicron transition	W15 (2022)	W52 (2022)	Period of declining transmission after Omicron, with intermittent smaller outbreaks, continued service adaptation, booster campaigns, and progressive relaxation of public health restrictions.
6	Post-pandemic period (endemic transition and routine management)	W01 (2023)	W52 (2024)	Period in which COVID-19 was increasingly managed as an endemic condition, with routine surveillance and integration into standard care; includes the WHO May 2023 declaration ending the PHEIC status.

**Figure 1:**
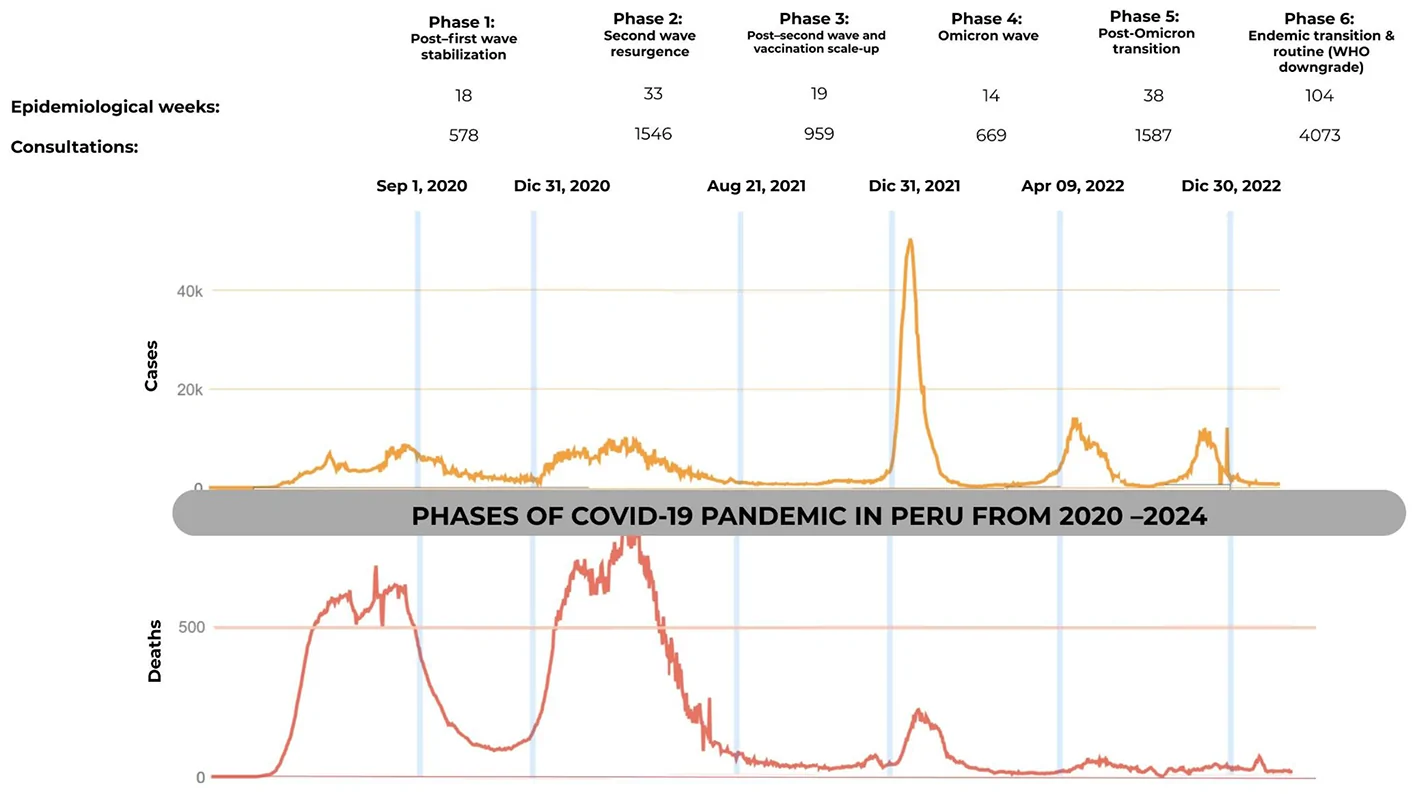
COVID-19 pandemic phases in Peru, 2020–2024. The COVID-19 case and mortality charts were adapted from the Johns Hopkins University Coronavirus Resource Center (23). Phase classifications, relevant dates, epidemiological weeks, and consultation counts were added by the authors. Adapted from Johns Hopkins University and Medicine (23).

### Outcomes

The primary outcome was the weekly psychotropic prescription rate per 1,000 consultations, referred to as the prescription rate. It was calculated as the ratio of the weekly psychotropic prescriptions to the total number of weekly psychiatric consultations, multiplied by 1,000, to standardize for variations in consultation volume. Because a single psychiatric consultation could result in the prescription of more than one psychotropic medication, each prescribed psychotropic drug was counted separately in the numerator. The epidemiological week constituted the unit of analysis for all primary analyses.

Psychotropic medications were classified according to the World Health Organization Anatomical Therapeutic Chemical (ATC) system (24): antipsychotics (N05A), antidepressants (N06A), anxiolytics (N05B), and mood stabilizers were operationally defined as lithium (N05AN01) and antiepileptic drugs commonly used as mood stabilizers (N03A).

As secondary outcomes, we assessed the number of consultations per epidemiological week and the annual proportion of patients receiving at least one psychotropic drug prescription, overall and stratified by medication class.

### Data processing

All records were pseudonymized prior to analysis. No linkage with external databases was required, as all information was contained within the institutional database.

Quality control of data was conducted to ensure internal consistency and analytical validity. Each consultation entry was independently reviewed by two researchers in two independent review rounds (JFSC and CFF). Records with missing or invalid dates, duplicated entries, or implausible values were excluded.

Each psychiatric consultation was recorded with a specific date and was subsequently aggregated into consecutive epidemiological weeks, which constituted the primary temporal unit of analysis. Then, for each epidemiological week, the total number of psychotropic prescriptions and the total number of consultations were calculated. Finally, each COVID-19 phase was operationalized as contiguous blocks of epidemiological weeks.

### Statistical analysis

Quantitative variables are represented as medians with interquartile ranges (IQR), and qualitative data as frequencies. Annual periods were used to describe patient characteristics, pandemic vs. post-pandemic periods were used for global descriptive comparisons, and the six predefined COVID-19 phases constituted the primary analytical framework for ITS analyses. Given the non-normal distribution of the data, non-parametric tests were applied. Comparisons between the pandemic and post-pandemic periods were carried out using the Mann–Whitney *U* test. Differences across the six COVID-19 phases were assessed using the Kruskal–Wallis test, followed by Dunn's *post hoc* test with Holm-adjusted *p*-values for multiple pairwise comparisons.

The temporal evolution and changes in trends of psychotropic prescriptions across the different study phases were analyzed using an ITS approach. Initially, an exploratory analysis was conducted through graphical visualization of the weekly time series over the entire study period. For descriptive purposes only, smoothing was applied using a 3-week moving average within each phase to identify overall patterns and local trends. The weekly time series comprised consecutive epidemiological weeks with no missing observations; therefore, no imputation or interpolation was required.

Subsequently, a single segmented regression model was fitted using generalized least squares (GLS), incorporating a first-order autoregressive [AR (1)] correlation structure in the residuals to account for serial dependence between consecutive observations. The residual correlation structure was selected after comparing models with independent, AR (1), and ARMA (1, 1) correlation structures using the Akaike Information Criterion (AIC) and Bayesian Information Criterion (BIC; Supplementary Table S1). The model was fitted jointly across the entire weekly time series and included a continuous time variable to estimate the baseline trend, indicator variables representing each phase to assess immediate level changes, and interaction terms between time and phase to estimate changes in slope across consecutive phases.

Immediate level changes and changes in trend between consecutive phases were estimated from the corresponding coefficients of the segmented regression model. Results are reported as regression coefficients (β) with 95% confidence intervals (95% CI) and *p*-values. Statistical significance was assessed using Wald *t*-tests for the corresponding regression coefficients.

All statistical tests were conducted at a 5% significance level. Analyses were conducted using R software (version 4.5.2; R Foundation for Statistical Computing, Vienna, Austria).

## Results

### Baseline characteristics of patients and annual psychotropic drug prescriptions

A total of 6,151 hospitalized patients were included in this study (the patient selection process is described in the Supplementary Figure 1). We found 384, 1,557, 1,434, 1,351, and 1,425 patients for each year from 2020 to 2024, respectively.

During the study period, the proportion of older adults ranged from 39.1% to 43.7%. Women represented more than half of the population (55.2%−61.3%). The proportion of patients who received a psychiatric diagnosis during hospitalization remained stable over time (97.1%−99.1%). A detailed distribution of psychiatric diagnoses (pre-existing and those identified during hospitalization) is presented in Supplementary Table 2.

The proportion of patients receiving at least one psychotropic medication ranged from 84.8% to 91.7% across the study years. Anxiolytics were the most frequently prescribed drug from 2020 to 2023 (52.1%, 51.0%, 48.9%, and 48.6%, respectively), whereas in 2024, antidepressants became the most commonly prescribed psychotropic subclass (45.9%). Detailed annual distributions of psychotropic prescriptions and baseline characteristics of patients are provided in Table 2.

**Table 2:** Demographic characteristics and psychotropic drug prescriptions of hospitalized Peruvian patients who received psychiatric medical care in a general hospital during the period 2020 and 2024.

	2020		2021		2022		2023		2024	
Characteristic	Set–Dec		Jan–Dec		Jan–Dec		Jan–Dec		Jan - Dec	
	*n*	*%*	*n*	*%*	*n*	*%*	*n*	%	*n*	%
Population	384		1557		1434		1351		1425	
Age (median [IQR])	59	[29]	60	[26]	61	[27]	61	[27]	60	[28]
Older adults (≥65)	150	39.1	625	40.1	612	42.7	590	43.7	586	41.1
Female	215	56	860	55.2	838	58.4	821	60.8	874	61.3
Pre-existing psychiatric diagnosis	126	32.8	408	26.2	413	28.8	427	31.6	508	35.6
Psychiatric diagnosis identified during hospitalization	377	98.2	1543	99.1	1414	98.6	1327	98.2	1384	97.1
Patient with at least one psychotropic drug prescription	331	86.2	1411	90.6	1230	85.8	1239	91.7	1208	84.8
Antipsychotic drug prescription	125	32.6	583	37.4	419	29.2	436	32.3	394	27.6
Antidepressant drug prescription	181	47.1	706	45.3	565	39.4	639	47.3	654	45.9
Mood stabilizer drug prescription	23	6	68	4.4	60	4.2	62	4.6	53	3.7
Anxiolytic drug prescription	200	52.1	794	51	701	48.9	657	48.6	552	38.7

IQR, interquartile range.

Additional information about the annual number of psychiatric consultations and the absolute number of psychotropic prescriptions by drug class is provided in Supplementary Table 3.

### Psychotropic drug prescription during and after the COVID-19 pandemic

The overall psychotropic prescription rate was significantly higher in the post-pandemic period compared with the COVID-19 pandemic period (median [IQR]: 1,308 [240] vs. 1,254 [197], *p* = 0.023, respectively). A similar pattern was observed for antidepressants [500 (106) vs. 403(133), *p* < 0.0001, respectively]. In contrast, anxiolytic prescriptions were significantly lower after the pandemic than during it [413 (157) vs. 444 (119), *p* = 0.048, respectively]. Antipsychotic and mood stabilizer prescription rates did not show statistically significant differences between these periods (*p* > 0.05 for both).

Finally, we found that the number of consultations per epidemiological week during the post-pandemic period was lower than the pandemic period (38 [13] vs. 44 [15], respectively, *p* < 0.001; Table 3).

**Table 3:** Epidemiological-week consultation counts and psychotropic prescription rates per 1,000 consultations during and after the COVID-19 pandemic.

Variable	Pandemic period	Post-pandemic period	
	2020–2022	2023–2024	*p* value
Number of epidemiological weeks	122	104	
Number of consultations	5339	4073	
Number of consultations per epidemiological week (median [IQR])	44 [15]	38 [13]	< 0.001
Prescription rates (median [IQR])			
Overall psychotropics	1254 [197]	1308 [240]	0.023
Antipsychotics	350 [157]	343 [140]	0.627
Antidepressants	403 [133]	500 [106]	< 0.0001
Mood stabilizers	36 [46]	34 [65]	0.882
Anxiolytics	444 [119]	413 [157]	0.048

Mann–Whitney *U* test. IQR, interquartile range. Prescription rates were calculated as the ratio of the weekly psychotropic prescriptions to the total number of weekly psychiatric consultations, multiplied by 1000.

### Psychotropic drug prescription across the COVID-19 pandemic phases

Prescription rates differed significantly across the six COVID-19 phases for overall psychotropics (*p* < 0.001), antipsychotics (*p* < 0.0001), antidepressants (*p* < 0.0001), and anxiolytics (*p* = 0.03), whereas mood stabilizer prescribing did not differ significantly across phases (*p* = 0.81). Number of consultations per epidemiological week also differed significantly among phases (*p* < 0.0001).

Detailed *post hoc* pairwise comparisons supporting these findings are presented in Table 4 and Supplementary Figure 2.

**Table 4:** Epidemiological-week consultation counts and psychotropic prescription rates per 1,000 consultations across the pandemic and post-pandemic phases.

Variable	Phase 1	Phase 2	Phase 3	Phase 4	Phase 5	Phase 6
	2020: W36 – W53	2021: W01 –W33	2021: W34–W52	2022: W01–W14	2022: W15–W52	2023: W1–2024: W52
Number of weeks	18	33	19	14	38	104
Total number of consultations	578	1546	959	669	1587	4073
Number of consultations per epidemiological week (median [IQR])	32 [6]	46 [12]^a****,d**^	52 [14]^a****,d*****, c*^	50 [13]^a***, d*^	42 [7]^a**^	38 [13]^a*^
Prescription rates (median [IQR])						
Overall psychotropics	1254 [145]	1318 [167]^c***^	1310 [168]	1207 [195]	1199 [239]	1308 [240]^c****^
Antipsychotics	385 [140]^c*^	429 [108]	288 [107]^b**^	366 [99]	296 [121]^b****^	343 [140]^b**^
Antidepressants	403 [192]	420 [102]^d**^	429 [93]	384 [123]^d*^	370 [116]^d****^	500 [106]
Mood stabilizers	31 [49]	32 [47]	32 [21]	38 [37]	49 [68]	33 [65]
Anxiolytics	432 [155]	444 [144]	490 [84]^d*^	417 [44]	448 [112]	413 [157]

Kruskal–Wallis test with Dunn's *post-hoc* test. Superscript letters indicate significant pairwise comparisons: ^a^= vs. Phase 1; ^b^= vs. Phase 2; ^c^= vs. Phase 5; ^d^= vs. Phase 6.

Asterisks indicate the level of significance: ^*^p < 0.05; ^**^p < 0.01; ^***^p < 0.001; ^****^p < 0.0001.

IQR, interquartile range. Prescription rates were calculated as the ratio of the weekly psychotropic prescriptions to the total number of weekly psychiatric consultations, multiplied by 1000.

### Psychotropic prescribing trends across the COVID-19 pandemic phases

The temporal patterns of psychotropic drug prescription rates and consultation varied across pandemic phases, reflecting a dynamic impact of COVID-19 on mental health care utilization in this Peruvian population (Figure 2).

**Figure 2:**
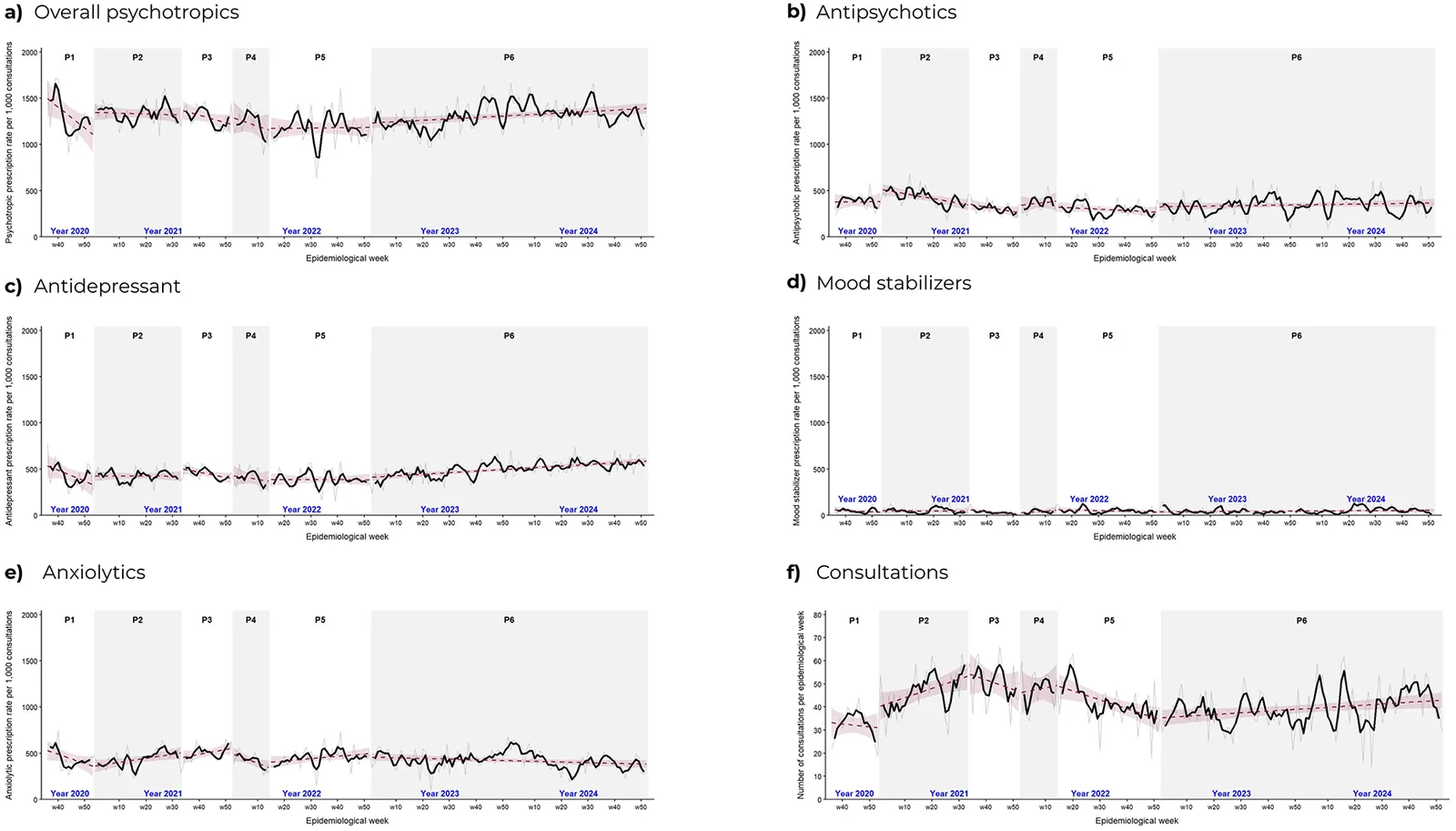
Trends in psychotropic drug prescribing, and consultation volumes across the six phases of the COVID-19 pandemic in Peru. **(a)** Overall psychotropics. **(b)** Antipsychotics. **(c)** Antidepressants. **(d)** Mood stabilizers. **(e)** Anxiolytics. **(f)** Consultations. Thin gray lines represent observed weekly values, solid black lines denote three-week moving averages computed within each pandemic phase, and dashed maroon lines indicate phase-specific linear trends with their corresponding 95% confidence intervals shown as shaded bands.

Prescription rate of overall psychotropics exhibited a significant downward trend during phase 1 (β = −25.53; 95% CI = −43.82 to −7.01; *p* = 0.007), followed by a clear structural break in phase 2, characterized by an abrupt level increase (β = 295.53; 95% CI = 60.48 to 530.57; *p* = 0.014) and a significant reversal in trend (β = 23.79; 95% CI = 3.33 to 44.26; *p* = 0.016). In the subsequent phases, including the post-pandemic period, no significant changes in slopes were observed, and prescription rates remained relatively stable (*p* > 0.05 for all comparisons).

Antidepressant prescriptions followed a similar pattern, with a significant decline in phase 1 (β = −12.28; 95% CI: −21.04 to −3.52; *p* = 0.006), and a structural break in phase 2 driven by a change in trend (β = 12.13; 95% CI: 2.64 to 21.62; *p* = 0.011). The following phases showed no consistent trend modifications (*p* > 0.05). In contrast, anxiolytic prescription rates exhibited a more dynamic pattern, with an initial marginal downward trend in phase 1 (β = −10.05; 95% CI = −21.27 to 1.18; *p* = 0.074), a significant positive change in trend during phase 2 (β = 14.15; 95% CI = 1.80 to 26.50; *p* = 0.01), and a subsequent marginal decrease in trend during phase 6 (β =-3.56; 95% CI = −7.44 to 0.32; *p* = 0.072). Antipsychotic and mood stabilizer prescriptions remained largely stable during the pandemic and post-pandemic periods (*p* > 0.05 for all comparisons).

Finally, the number of consultations per week displayed a marginal abrupt increase in phase 2 (β =10.95; 95% CI = −0.32 to 22.22; *p* = 0.057), followed by a sustained decline throughout phases 3 to 5. In the post-pandemic phase, a significant positive change in trend was observed (β = 0.44; 95% CI = 0.15 to 0.73; *p* = 0.003), suggesting a partial recovery in mental health service utilization. Full model coefficients, 95% CI and phase-specific slope dynamics are reported in Supplementary Table 4.

## Discussion

Our initial results indicate that, compared with the COVID-19 pandemic period, the post-pandemic period showed higher prescription rates for overall psychotropics and antidepressants, stable rates for antipsychotics and mood stabilizers, and lower rates for anxiolytics, alongside a reduction in the number of consultations per epidemiological week. These results suggest that the transition between periods may reflect changes in prescribing dynamics rather than a simple return to pre-pandemic patterns.

The higher overall psychotropic prescription rate observed during the post-pandemic period was accompanied by a substantial increase in antidepressant prescribing, whereas the other psychotropic subclasses remained relatively stable. Although this pattern could be associated with changes in the burden of depressive disorders during the post-pandemic period (25–27), this interpretation should be made cautiously. In Peru, most available post-pandemic evidence comes from cross-sectional surveys of the general population using self-reported instruments (25–27), which may not adequately capture healthcare utilization patterns or the actual demand for psychiatric care. Other factors may also have contributed to the observed increase in antidepressant prescribing, including shifts in the profile of hospitalized patients or in prescribing practices within the consultation-liaison service, although these factors were not directly evaluated in the present work.

In our study, the number of psychiatric consultations per epidemiological week decreased during the post-pandemic period. Because this study was conducted in a consultation-liaison psychiatry service, consultation volume likely reflects not only the need for psychiatric care but also referral practices from other hospital departments. For this reason, the reduction in consultations should not be interpreted simply as evidence of lower psychiatric morbidity. It may also reflect broader post-pandemic changes in hospital dynamics, such as variations in hospitalization patterns or in the threshold for requesting psychiatric consultation (28, 29). These contextual factors were beyond the scope of the present study, but they should be considered when interpreting the findings.

Notably, antidepressant prescription rates increased during the post-pandemic period despite the reduction in psychiatric consultations. Although the reasons underlying this pattern cannot be determined from the present study, one possible explanation is that, following the pandemic period, psychiatrists may have had more opportunities to reassess patients, initiate or adjust antidepressant treatments, and optimize pharmacological management during consultations (10, 11, 30). In this context, the observed increase in antidepressant prescribing may not be explained solely by changes in the burden of depressive disorders, but may also reflect changes in the clinical management of patients receiving psychiatric care within the consultation-liaison service (10, 11, 30).

Exploratory phase-level comparisons across the six pandemic phases exhibited significant differences in most psychotropic subclasses. Consistent with the comparisons between COVID-19 and post-pandemic periods described above, overall psychotropic and antidepressant prescriptions were higher in phase 6, while the number of consultations declined relative to the earlier pandemic phases. A similar scenario was reported in Italy (14), where a reduction in psychiatric consultations was not accompanied by a proportional decrease in antidepressant prescribing (14).

In contrast, the anxiolytic prescription rate was lower in phase 6 compared with the intermediate phase; antipsychotics demonstrated greater phase-specific variability, and mood stabilizer prescribing remained stable across pandemic phases. These heterogeneous changes are comparable to those reported in HICs (15, 16, 31), where psychotropic subclasses exhibited differential responses over time rather than a uniform prescribing pattern. Nonetheless, these findings should be interpreted cautiously, as these phase-level comparisons identify distributional differences across predefined periods without accounting for temporal autocorrelation or pre-existing trends.

In the ITS analysis, we observed a downward trend in the prescription rate of overall psychotropics during phase 1. Because the study period began after the first COVID-19 wave in Peru, this finding may represent the continuation of a pre-existing secular trend or reflect reduced access to psychiatric care and changes in healthcare utilization following the first pandemic wave. This was followed by a clear structural break in phase 2, marked by an abrupt level increase and significant slope reversal, suggesting changes in psychiatric prescribing patterns during the second pandemic wave in Peru. These findings are partially consistent with Huang *et al*. (21), who reported an abrupt increase in psychotropic consumption during the first quarter of the pandemic, followed by a significant attenuation in long-term growth trends. However, their analyses were performed using aggregated national sales data, which could have been influenced by stockpiling in the initial phases of the COVID-19 pandemic. In contrast, our data reflect real-world clinical demand among hospitalized patients. Moreover, the structural rebound observed in our study is consistent with the “shock-correction-adaptation” sequence detailed in multinational studies (20, 21). These findings suggest that in MICs with historically low baseline consumption (20, 21), service disruption may initially suppress prescribing before stabilization occurs.

Class-specific differences were also observed. Among the psychotropic subclasses, antidepressant prescription rates showed the temporal pattern most similar to that observed for overall psychotropic prescribing. In contrast, anxiolytic prescription rates exhibited a short-term volatility across the study phases, with no clear long-term pattern. Our more granular six-phase segmentation may also explain part of the heterogeneity reported in prior studies that relied on broader pre/during/post classifications (13, 15, 16, 31).

On the other hand, antipsychotic and mood stabilizer prescription rates remained relatively stable across the study period. This relative stability may reflect the clinical necessity of maintaining pharmacological treatment for severe psychiatric and neuropsychiatric conditions even during systemic health disruption. Alternatively, it may also be related to the consultation-liaison setting of our study, in which these medications may be used in hospitalized patients with neuropsychiatric manifestations associated with acute medical illness, some of which may be less sensitive to pandemic-related fluctuations than primary psychiatric disorders, such as delirium or organic psychoses (32, 33). However, prescribing indications were not evaluated in the present study, and this interpretation should therefore be considered cautiously.

This study has some limitations. First, the study period began during the post-first wave stabilization phase of the COVID-19 pandemic in Peru and therefore did not capture the first pandemic wave. Consequently, our ITS analysis should be interpreted as describing temporal changes across predefined pandemic phases rather than the entire course of the pandemic. In addition, because the study began in September 2020 (late winter in the Southern Hemisphere), potential seasonal influences on psychiatric consultations and prescribing patterns could not be explicitly assessed. Therefore, part of the initial downward trend observed during Phase 1 may reflect seasonal variation or continuation of pre-existing trends rather than pandemic-related changes alone. Second, the study relied on data from a single tertiary-care hospital within the EsSalud healthcare system and included only hospitalized patients, who generally present with greater clinical severity. Therefore, the findings may not be generalizable to community mental health centers, private healthcare institutions, or other healthcare settings in Peru. Additionally, we did not evaluate individual-level adherence or long-term clinical outcomes. Finally, the relatively short duration of Phase 4 (14 epidemiological weeks) may have reduced the precision of the estimated level and trend changes, as reflected by the wider confidence intervals. Accordingly, findings for this phase should be interpreted with caution.

Despite these limitations, the study has important strengths. It provides one of the few ITS analyses of psychotropic prescribing in a Latin American population (13), a region underrepresented in the literature. By using real-world inpatient prescription data, our findings reflect actual clinical demand in a vulnerable population, offering a complementary perspective to national sales-based studies that rely on aggregated data (20, 21).

In conclusion, our study reveals dynamic psychotropic prescribing trends in Peru during and after the COVID-19 pandemic. Overall psychotropic prescribing showed phase-specific disruption followed by structural rebound and stabilization, while antidepressant prescribing showed a similar temporal pattern. These findings provide real-world evidence from an underrepresented Latin American setting and underscore the need to monitor psychiatric prescribing and mental health service adaptation in resource-limited settings.

## Data Availability

The de-identified data supporting the findings of this study are available from the corresponding author upon reasonable request, subject to applicable ethical and institutional requirements.
