# Supplementary material for: Psychotropic prescribing trends in a Peruvian General Hospital during and after the COVID-19 pandemic: an interrupted time-series analysis
Source: Front Public Health. 2026 Oct 9;14:1892034. doi: 10.3389/fpubh.2026.1892034 (PMC13693525; doi:10.3389/fpubh.2026.1892034)
Supplement: Supplementary file 1 [file Supplementary_file_1.docx]

Supplementary Material

# 1 Supplementary Table

**Supplementary Table S1.** Comparison of residual correlation structures for the segmented GLS model.

| **Correlation structure** | **Parameters** | **AIC** | **BIC** |
| --- | --- | --- | --- |
| Independent | 13 | 2856.87 | 2900.63 |
| AR(1) | 14 | 2835.80 | 2882.93 |
| ARMA(1,1) | 15 | 2837.25 | 2887.74 |

AR(1) = First-order autoregressive model; ARMA(1,1) = First-order autoregressive moving-average model; AIC – Akaike Information Criterion; BIC – Bayesian Information Criterion; GLS – Generalized least squares

**Supplementary Table 2**. Distribution of psychiatric diagnoses (ICD-10) before and during medical evaluation, by year (2020–2024).

|  | **2020** | | **2021** | | **2022** | | **2023** | | **2024** | |
| --- | --- | --- | --- | --- | --- | --- | --- | --- | --- | --- |
|  | **n=384** | | **n=1557** | | **n=1434** | | **n=1351** | | **n=1425** | |
|  | n | % | n | % | n | % | n | % | n | % |
| **Pre-existing psychiatric diagnosis** |  |  |  |  |  |  |  |  |  |  |
| Organic mental disorders (F00-F09) | 17 | 4.4 | 44 | 2.8 | 46 | 3.2 | 47 | 3.5 | 51 | 3.6 |
| Mental disorders due to substance use (F10 - F19) | 0 | 0 | 10 | 0.6 | 5 | 0.3 | 10 | 0.7 | 17 | 1.2 |
| Schizophrenia spectrum disorders (F20 - F29) | 12 | 3.1 | 43 | 2.8 | 20 | 1.4 | 25 | 1.9 | 26 | 1.8 |
| Mood disorders (F30 - F39) | 52 | 13.5 | 163 | 10.5 | 217 | 15.1 | 199 | 14.7 | 205 | 14.4 |
| Anxiety, stress-related, and somatoform disorders (F40 - F48) | 35 | 9.1 | 120 | 7.7 | 106 | 7.4 | 130 | 9.6 | 179 | 12.6 |
| Syndromes associated with physiological disturbances (F50-F59) | 6 | 1.6 | 16 | 1 | 10 | 0.7 | 8 | 0.6 | 11 | 0.8 |
| Personality disorders (F60 - F69) | 1 | 0.3 | 6 | 0.4 | 4 | 0.3 | 3 | 0.2 | 13 | 0.9 |
| Intellectual disability (F70 - F79) | 2 | 0.5 | 2 | 0.1 | 2 | 0.1 | 4 | 0.3 | 3 | 0.2 |
| Disorders of psychological development (F80 - F89) | 1 | 0.3 | 2 | 0.1 | 0 | 0 | 0 | 0 | 0 | 0 |
| Childhood and/or adolescence disorders (F90 - F98) | 0 | 0 | 2 | 0.1 | 3 | 0.2 | 1 | 0.1 | 3 | 0.2 |
| None | 258 | 67.19 | 1149 | 73.8 | 1021 | 71.2 | 924 | 68.4 | 917 | 64.6 |
| **Received a psychiatric diagnosis during medical evaluation** |  |  |  |  |  |  |  |  |  |  |
| Organic mental disorders (F00 - F09) | 82 | 21.4 | 371 | 23.8 | 318 | 22.2 | 292 | 21.6 | 261 | 18.3 |
| Mental disorders due to substance use (F10 - F19) | 1 | 0.3 | 8 | 0.5 | 9 | 0.6 | 3 | 0.2 | 11 | 0.8 |
| Schizophrenia spectrum disorders (F20 - F29) | 16 | 4.2 | 38 | 2.4 | 28 | 2 | 27 | 2 | 27 | 1.9 |
| Mood disorders (F30 - F39) | 82 | 21.4 | 354 | 22.7 | 355 | 24.8 | 302 | 22.4 | 389 | 27.3 |
| Anxiety, stress-related, and somatoform disorders (F40 - F48) | 185 | 48.2 | 723 | 46.4 | 653 | 45.5 | 655 | 48.5 | 558 | 39.2 |
| Syndromes associated with physiological disturbances (F50 - F59) | 4 | 1 | 38 | 2.4 | 29 | 2 | 34 | 2.5 | 120 | 8.4 |
| Personality disorders (F60 - F69) | 6 | 1.6 | 8 | 0.5 | 14 | 1 | 9 | 0.7 | 15 | 1.1 |
| Intellectual disability (F70 - F79) | 1 | 0.3 | 2 | 0.1 | 4 | 0.3 | 5 | 0.4 | 3 | 0.2 |
| Disorders of psychological development (F80 - F89) | 0 | 0 | 0 | 0 | 3 | 0.2 | 0 | 0 | 0 | 0 |
| Childhood and/or adolescence disorders (F90 - F98) | 0 | 0 | 1 | 0.1 | 1 | 0.1 | 0 | 0 | 0 | 0 |
| None | 7 | 1.82 | 14 | 0.9 | 20 | 1.39 | 24 | 1.8 | 41 | 2.9 |

**Supplementary Table 3**. Annual number of consultations, epidemiological weeks, and absolute psychotropic prescriptions by drug class (2020–2024).

|  | 2020 | | 2021 | | 2022 | | 2023 | | 2024 | |
| --- | --- | --- | --- | --- | --- | --- | --- | --- | --- | --- |
|  | Set-Dec | | Jan - Dec | | Jan - Dec | | Jan - Dec | | Jan - Dec | |
|  | N | % | N | % | N | % | N | % | N | % |
| Number of Consultation | 578 | | 2505 | | 2256 | | 1924 | | 2149 | |
| Number of Epidemiological weeks | 18 | | 52 | | 52 | | 52 | | 52 | |
| Absolute Number of Prescriptions per Drug |  |  |  |  |  |  |  |  |  |  |
| Psychotropics | 750 | | 3302 | | 2678 | | 2435 | | 2924 | |
| Antipsychotic | 224 | 29.87 | 951 | 28.8 | 714 | 26.66 | 642 | 26.37 | 795 | 27.18 |
| Antidepressant | 248 | 33.07 | 1091 | 33.04 | 872 | 32.56 | 884 | 36.3 | 1157 | 39.56 |
| Mood stabilizers | 27 | 3.6 | 106 | 3.21 | 107 | 4 | 71 | 2.92 | 105 | 3.5 |
| Anxiolytic | 251 | 33.47 | 1154 | 34.95 | 985 | 36.78 | 838 | 34.41 | 867 | 29.65 |

**Supplementary Table 4**. Immediate changes in level, changes in trend, and slope dynamics by phase of the COVID-19 in Peru.

|  | **Immediate level change** | | | | **Trend changes** | | | | **Full dynamic interpretation** | | |
| --- | --- | --- | --- | --- | --- | --- | --- | --- | --- | --- | --- |
| **Phase** | **Parameter** | **Coef (95% CI)** | **p** | **Interpretation** | **Comparison** | **Coef (95% CI)** | **p** | **Interpretation** | **Slope per phase** | **Value** | **Interpretation** |
| **Psychotropic**: |  |  |  |  |  |  |  |  |  |  |  |
| - | Intercept | 1520.42 (1332.47 to 1708.37) | <0.001 | Estimated level at the first observed epidemiological week | - | - |  |  |  |  |  |
| Phase 1 | P1 (basal) | -25.53 (-43.82 to -7.01) | 0.007 | Decrease of 25.4 units per week | - | - |  |  | F1 | 25.41 | Phase 1: Significant downward trend |
| Phase 2 | P2 | 295.53 (60.48 to 530.57) | 0.014 | Abrupt increase | T2 (F2 vs F1) | 23.79 (3.33 to 44.26) | 0.023 | Strong trend change | F2 | -1.62 | Phase 2: Significant clear structural break |
| Phase 3 | P3 | 55.87 (−167.93 to 279.67) | 0.623 | No immediate change | T3 (F3 vs F2) | −4.16 (−23.23 to 14.91) | 0.668 | No change | F3 | -5.78 | Phase 3: Stable |
| Phase 4 | P4 | −21.61 (−283.44 to 240.22) | 0.871 | No immediate change | T4 (F4 vs F3) | 2.32 (−29.95 to 34.60) | 0.887 | No change | F4 | -3.46 | Phase 4: Stable |
| Phase 5 | P5 | −16.96 (−266.73 to 232.81) | 0.894 | No immediate change | T5 (F5 vs F4) | 4.57 (−22.50 to 31.65) | 0.74 | No change | F5 | 1.11 | Phase 5: Stable |
| Phase 6 | P6 | 21.16 (−140.22 to 182.54) | 0.796 | No immediate change | T6 (F6 vs F5) | 0.46 (−6.00 to 6.92) | 0.888 | No change | F6 | 1.57 | Phase 6: Stable |
|  |  |  |  |  |  |  |  |  |  |  |  |
| **Antipsychotic:** |  |  |  |  |  |  |  |  |  |  |  |
| - | Intercept | 362.85 (242.37 to 483.34) | <0.001 | Estimated level at the first observed epidemiological week | - | - | - |  |  |  |  |
| Phase 1 | P1 (basal) | 1.51 (-10.31 to 13.33) | 0.801 | No immediate change | - | - | - |  | F1 | 1.51 | Phase 1: Stable |
| Phase 2 | P2 | 119.45 (-31.82 to 270.72) | 0.121 | No immediate change | T2 (F2 vs F1) | -6.54 (-19.65 to 6.58) | 0.33 | No change | F2 | −5.03 | Phase 2: Stable |
| Phase 3 | P3 | 0.88 (-143.12 to 144.88) | 0.99 | No immediate change | T3 (F3 vs F2) | 1.14 (-11.08 to 13.37) | 0.854 | No change | F3 | −5.55 | Phase 3: Stable |
| Phase 4 | P4 | 73.99 (-94.91 to 242.89) | 0.389 | No immediate change | T4 (F4 vs F3) | 5.52 (-15.19 to 26.24) | 0.6 | No change | F4 | 9.96 | Phase 4: Stable |
| Phase 5 | P5 | -40.38 (-201.29 to 120.53) | 0.621 | No immediate change | T5 (F5 vs F4) | -3.12 (-20.52 to 14.28) | 0.724 | No change | F5 | 0.24 | Phase 5: Stable |
| Phase 6 | P6 | 52.25 (-51.22 to 155.71) | 0.321 | No immediate change | T6 (F6 vs F5) | 1.91 (-2.23 to 6.05) | 0.364 | No change | F6 | 1.52 | Phase 6: Stable |
|  |  |  |  |  |  |  |  |  |  |  |  |
| **Antidepressant**: |  |  |  |  |  |  |  |  |  |  |  |
| - | Intercept | 538.37 (450.84 to 625.90) | <0.001 | Estimated level at the first observed epidemiological week | - | - | - |  |  |  |  |
| Phase 1 | P1 (basal) | -12.28 (-21.04 to -3.52) | 0.006 | Significant decrease of 12.28 units per week | - | - | - |  | F1 | −12.28 | Phase 1: Significant downward trend |
| Phase 2 | P2 | 109.85 (-5.11 to 224.81) | 0.061 | Marginal abrupt increase | T2 (F2 vs F1) | 12.13 (2.64 to 21.62) | 0.012 | Significant upward change in trend | F2 | −0.15 | Phase 2: Significant structural break |
| Phase 3 | P3 | 75.94 (-33.16 to 185.05) | 0.172 | No immediate change | T3 (F3 vs F2) | -5.25 (-14.12 to 3.61) | 0.244 | No significant change in trend | F3 | −5.40 | Phase 3: Stable |
| Phase 4 | P4 | 26.53 (-106.32 to 159.38) | 0.694 | No immediate change | T4 (F4 vs F3) | 1.79 (-13.39 to 16.97) | 0.816 | No significant change in trend | F4 | −3.61 | Phase 4: Stable |
| Phase 5 | P5 | 13.66 (-110.48 to 137.80) | 0.829 | No immediate change | T5 (F5 vs F4) | 3.63 (-9.45 to 16.70) | 0.585 | No significant change in trend | F5 | 0.02 | Phase 5: Stable |
| Phase 6 | P6 | 25.68 (-48.96 to 100.32) | 0.498 | No immediate change | T6 (F6 vs F5) | 1.69 (-1.26 to 4.64) | 0.259 | No significant change in trend | F6 | 1.71 | Phase 6: Stable |
|  |  |  |  |  |  |  |  |  |  |  |  |
| **Mood Stabilizers:** |  |  |  |  |  |  |  |  |  |  |  |
| - | Intercept | 60.95 (16.36 to 105.53) | 0.008 | Estimated level at the first observed epidemiological week | - | - | - |  |  |  |  |
| Phase 1 | P1 (basal) | −1.84 (−6.27 to 2.58) | 0.413 | Stable baseline trend | - | - | - |  | F1 | −1.84 | Phase 1: Stable |
| Phase 2 | P2 | 22.59 (−34.98 to 80.16) | 0.44 | No immediate change | T2 (F2 vs F1) | 1.71 (−3.14 to 6.55) | 0.489 | No change | F2 | −0.14 | Phase 2: Stable |
| Phase 3 | P3 | 15.83 (−38.89 to 70.55) | 0.569 | No immediate change | T3 (F3 vs F2) | −3.08 (−7.61 to 1.44) | 0.181 | No change | F3 | −3.22 | Phase 3: Stable |
| Phase 4 | P4 | 26.05 (−39.46 to 91.57) | 0.435 | No immediate change | T4 (F4 vs F3) | 5.41 (−2.31 to 13.12) | 0.169 | No change | F4 | 2.19 | Phase 4: Stable |
| Phase 5 | P5 | 3.74 (−58.02 to 65.49) | 0.905 | No immediate change | T5 (F5 vs F4) | −2.95 (−9.52 to 3.62) | 0.376 | No change | F5 | −0.76 | Phase 5: Stable |
| Phase 6 | P6 | 6.29 (−31.94 to 44.51) | 0.746 | No immediate change | T6 (F6 vs F5) | 0.90 (−0.62 to 2.41) | 0.246 | No change | F6 | 0.13 | Phase 6: Stable |
|  |  |  |  |  |  |  |  |  |  |  |  |
| **Anxiolytic**: |  |  |  |  |  |  |  |  |  |  |  |
| - | Intercept | 531.67 (418.13 to 645.22) | <0.001 | Estimated level at the first observed epidemiological week | - | - | - |  |  |  |  |
| Phase 1 | P1 (baseline) | −10.05 (−21.27 to 1.18) | 0.079 | Marginal abrupt downward | - | - | - |  | F1 | −10.05 | Phase 1: Stable |
| Phase 2 | P2 | 13.71 (−131.57 to 159.00) | 0.853 | No immediate change | T2 (F2 vs F1) | 14.15 (1.80 to 26.50) | 0.025 | Significant upward trend change | F2 | 4.1 | Phase 2: Significant structural break |
| Phase 3 | P3 | −40.05 (−178.21 to 98.12) | 0.568 | No immediate change | T3 (F3 vs F2) | 1.56 (−9.96 to 13.09) | 0.79 | No change | F3 | 5.66 | Phase 3: Stable |
| Phase 4 | P4 | −103.06 (−267.28 to 61.17) | 0.217 | No immediate change | T4 (F4 vs F3) | −13.24 (−32.85 to 6.37) | 0.185 | No change | F4 | −7.58 | Phase 4: Stable |
| Phase 5 | P5 | 40.43 (−114.95 to 195.82) | 0.609 | No immediate change | T5 (F5 vs F4) | 10.41 (−6.20 to 27.03) | 0.218 | No change | F5 | 2.84 | Phase 5: Stable |
| Phase 6 | P6 | −49.85 (−147.31 to 47.61) | 0.315 | No immediate change | T6 (F6 vs F5) | −3.56 (−7.44 to 0.32) | 0.072 | No change | F6 | −0.72 | Phase 6: Stable |
|  |  |  |  |  |  |  |  |  |  |  |  |
| **Consultation** |  |  |  |  |  |  |  |  |  |  |  |
| – | Intercept | 33.24 (24.61 to 41.88) | <0.001 | Estimated number of consultations at the first observed epidemiological week | - | - | - |  |  |  |  |
| Phase 1 | P1 (baseline) | −0.18 (−1.04 to 0.68) | 0.686 | Stable baseline trend | - | - | - |  | F1 | −0.18 | Phase 1: Stable |
| Phase 2 | P2 | 10.95 (−0.32 to 22.22) | 0.057 | Marginal abrupt increase | T2 (F2 vs F1) | 0.55 (−0.39 to 1.49) | 0.247 | No change | F2 | 0.37 | Phase 2: Stable |
| Phase 3 | P3 | 1.19 (−9.51 to 11.89) | 0.827 | No immediate change | T3 (F3 vs F2) | −0.84 (−1.72 to 0.03) | 0.059 | No change | F3 | −0.47 | Phase 3: Stable |
| Phase 4 | P4 | 1.24 (−11.71 to 14.18) | 0.851 | No immediate change | T4 (F4 vs F3) | 0.68 (−0.82 to 2.17) | 0.373 | No change | F4 | 0.21 | Phase 4: Stable |
| Phase 5 | P5 | −1.24 (−13.38 to 10.90) | 0.84 | No immediate change | T5 (F5 vs F4) | −0.58 (−1.86 to 0.71) | 0.376 | No change | F5 | −0.37 | Phase 5: Stable |
| Phase 6 | P6 | 0.83 (−6.56 to 8.21) | 0.825 | No immediate change | T6 (F6 vs F5) | 0.44 (0.15 to 0.73) | 0.003 | Significant upward trend change | F6 | 0.07 | Phase 6: Significant recovery in consultation trend |

## 2. Supplementary Figures

**Supplementary Figure 1.** Flowchart of the patient selection process

**Supplementary Figure 2.** Epidemiological-week consultation counts and psychotropic prescription rates per 1000 consultations across temporal phases of the COVID-19 pandemic in Peru (2020–2024).

Kruskal-Wallis test and *post-hoc* Dunn test. *Mean ± SEM.*

**a)** Psychotropics: vs Phase 5: ****p<0.0001; ***p<0.001. **b)** Antipsychotics: vs Phase 2: ****p<0.0001, **p<0.01; vs Phase 5: p<0.05. **c)** Antidepressant: vs Phase 6: ****p<0.0001, **p<0.01, *p<0.05. **e)** Anxiolytics: vs Phase 6: *p<0.05. **f)** Number of consultations per epidemiological week: vs Phase 1: ****p<0.0001, ***p<0.001, **p<0.01; vs Phase 6: ++++p<0.0001, ++p<0.01, +p<0.05, vs Phase 5. $p<0.05.
